# Assessment of Oxidative Stress by the Estimation of Lipid Peroxidation Marker Malondialdehyde (MDA) in Patients with Chronic Periodontitis: A Systematic Review and Meta-Analysis

**DOI:** 10.1155/2023/6014706

**Published:** 2023-05-30

**Authors:** Khadijah Mohideen, Krithika Chandrasekar, Saranya Ramsridhar, Chandini Rajkumar, Snehashish Ghosh, Safal Dhungel

**Affiliations:** ^1^Department of Oral Pathology and Microbiology, Sathyabama Dental College and Hospital, Sathyabama Institute of Science and Technology, Chennai 600119, India; ^2^Meenakshi Academy of Higher Education and Research, West K. K. Nagar, Chennai 600078, India; ^3^Department of Oral Pathology, College of Medical Sciences, Bharatpur 44200, Nepal; ^4^Department of Oral and Maxillofacial Surgery, College of Medical Sciences, Bharatpur 44200, Nepal

## Abstract

**Objective:**

The present systematic review and meta-analysis aimed to assess the oxidative stress-mediated lipid peroxidation end product malondialdehyde (MDA) in periodontitis using the available literature.

**Materials and Methods:**

An electronic literature search was performed for the published articles from 2000 to 2022 in PubMed (MeSH), Science Direct, Wiley Online library, and cross-reference using specific keywords.

**Results:**

The literature search identified 1,166 articles. After analyzing the abstracts of the obtained articles, the articles were excluded for the following reasons: duplicate studies (*n* = 395) and not relevant to the research question (*n* = 726). The remaining 45 articles were chosen for full-text evaluation. Finally, the present qualitative synthesis selected 34 articles that met the inclusion criteria for evaluation and removed the articles which did not meet the required criteria (*n* = 11). Out of these, 16 articles had coherent data for quantitative synthesis. The meta-analysis used the standardized mean differences method at a 95% confidence interval by random-effects model. The periodontitis group displayed significantly higher MDA levels (*P* < 0.001) in gingival crevicular fluid, saliva, and serum samples of the studies analyzed than the healthy control.

**Conclusion:**

The analyzed studies showed significantly higher MDA levels in various biological samples of patients with periodontitis, supporting the role of elevated oxidative stress and consequent lipid peroxidation in periodontitis.

## 1. Introduction

Periodontitis is one of the most widespread oral diseases, affecting around 50% of the adult population [1]. Its incidence differs in various populations and depends on an individual's oral hygiene and socioeconomic position [2]. The inflammatory chronic periodontal diseases are triggered by dental biofilm infection, leading to the destruction of supporting tissue and alveolar bone and teeth loss [3]. The progression of the disease is considered a complex interplay of exaggerated inflammatory reactions, genetic risk factors, smoking habits, poor oral hygiene, malnutrition, and aberrant immune responses caused by periodontal pathogens [4].

The reactive oxygen or nitrogen species (ROS/RNS) or free radicals are excessively produced mainly by hyperactive neutrophils or by direct microbial release, which cannot be counteracted by the antioxidant defense system and results in oxidative stress (OS) and subsequent tissue damage [5]. Low levels of ROS are essential to maintain critical biological processes, eliminating pathogenic micro-organisms and stimulating epithelial and fibroblast cell growth [6]. The elevated concentrations of ROS create an OS environment and promote a diversity of biological processes, such as neutrophil infiltration and activation of fibroblasts and osteoclasts [7]. It is established that OS is an integral part of the inflammatory process and modulates the burden of disease and healing. Independent of the mechanism, OS mediates pathologic effects, leading to cell membrane lysis, activation of proteolytic enzymes, inactivation of proteolytic enzyme inhibitors, deoxyribonucleic acid (DNA) fragmentation, and in most severe situations, cell death. Therefore, degradation of collagenous and extracellular matrix-specific components may occur, possibly explaining periodontal tissue destruction [8].

OS is characterized by increased metabolites or end products of lipid peroxidation (LPO), DNA damage, and protein damage [9]. Assessing these end products in various biological samples provides clues about specific footprints of lipid, protein, and DNA oxidative damage and is the most effective manner to detect OS response in a host. Optimal biomarkers for evaluating OS in pathological diseases should be stable, appreciable in small concentrations, reveal specific oxidation pathways, and relate to disease initiation, progression, and severity [10]. Though the free radicals or oxidants are highly reactive and possess a short half-life, the products released from the reaction of ROS with biological macromolecules are preferably used as biomarkers of oxidative injury in the pathological process of periodontitis [11].

LPO is the most commonly investigated derivative of oxidative damage associated with periodontal diseases [12]. LPO results from the reaction of lipids such as polyunsaturated fatty acids (PUFA) with RNS/ROS that results in a complex process of degradation or decomposition reactions of hydroperoxides and a wide range of end products are released [12]. The end products of LPO are more stable than free radicals. They can also react with other macromolecules far from their production site, including DNA, proteins, and phospholipids. The markers produced during LPO are malondialdehyde (MDA), 4-hydroxy-2-nonenal, Isoprostanes, and conjugated dienes. The most investigated LPO marker is MDA; MDA is released from fatty acids and consists of two or more methylene-interrupted double bonds [13]. Extensive research data indicates that the MDA level in various biological samples may be a reliable indicator of the extent of oxidative injury to cells and tissues of the body [14]. Available literature indicates that MDA levels are strongly linked with periodontal tissue inflammation and supporting tissue destruction [15]. Only a few studies dealt with other LPO markers, so the literature about their usefulness for monitoring oral diseases is limited.

It is essential and urgent to validate the OS-mediated LPO biomarkers' effectiveness and their association with periodontal disease. Thus, the present systematic review (SR) aims to analyze the literature about the level of OS-mediated LPO by assessing the mean value of MDA in biological samples of patients affected with periodontitis.

## 2. Materials and Methods

The present SR was performed per the PRISMA statement guidelines [16].

### 2.1. Focused Question of Interest

Is there a significant difference present in the levels of MDA between the patients affected with periodontitis and the healthy control group?

Considering the Population, Exposure, Comparators, and Outcomes principles, the SR focused on the alterations of OS-mediated LPO marker MDA in saliva, gingival crevicular fluid (GCF), serum, and plasma of patients with and without periodontitis to test the association between OS and periodontitis.

### 2.2. Electronic Search Identification

The literature search was performed in the electronic databases, including PubMed, Science Direct, Wiley online library and Cross-reference, for previously published articles that addressed lipid peroxidation in periodontitis by assessing MDA levels between 2000 and 2022 in the English language. The literature search in the PubMed database was performed using the following keywords in the title or abstract such as (1) keywords: ROS; Reactive oxygen species; OS; Oxidative stress; lipid peroxidation; MDA; Malondialdehyde; connected by Boolean operator OR. (2) keywords: Blood; saliva was connected by OR (3) keyword: Periodontitis was connected with AND. The articles identified in the PubMed database searches filtered by “humans”; publication year from 2000 to 2022; and articles in the English language.

Keywords searched in the Science Direct database were Oxidative Stress and Periodontitis. The articles identified in the Science Direct database searches filtered by Research Articles with subject areas: Medicine and Dentistry; Biochemistry, Genetics and Molecular Biology; Pharmacology, Toxicology and Pharmaceutical Science; publication year from 2000 to 2022.

In the Cochrane database, the title or abstract keywords given were lipid peroxidation AND periodontitis; Malondialdehyde AND periodontitis. The articles identified in the Cochrane database searches were filtered by publication year from 2000 to 2022; and articles in the English language.

In Wiley Online database, an adaptation of the below-mentioned search strategy was performed; <title>Wiley-Online-Library: keyword search (Keywords: Malondialdehyde) AND (Keywords: periodontitis) AND (Earliest: 2000 TO 2022).

### 2.3. Screening for Relevance

The titles and objectives of the identified articles were screened for relevance and duplication.

### 2.4. Inclusion Criteria

- Studies discussed the OS in periodontitis (localized or generalized) by the assessment of LPO marker MDA.
- Cross-sectional or prospective, case–control, and observational studies with a healthy control group were conducted in human adults without any restrictions on the sample size.
- Studies involving various biological samples and compared the data in the periodontitis group with the control group.

The case and control both included systemically healthy individuals who did not use antibiotics, anti-inflammatories, or other drugs and had no history of periodontal treatment in the last 3 months.

- The studies of systemic diseases associated with periodontitis included only if they included systemically healthy control and periodontitis group as a separate evaluation group for the specified marker assessment.
- The studies involving smokers associated with periodontitis included only when they included nonsmokers in periodontitis and the control group as a separate evaluation group for the specified marker assessment.
- The studies evaluated the specified marker after therapy effect in periodontitis included only if they specified baseline values for periodontitis and the control group in particular.

Papers provided MDA mean values and standard deviation (SD) or median values with (minimum–maximum) details along with statistical significance *P*-value.

### 2.5. Exclusion Criteria

- Studies that did not address periodontal disease lacked a control group and the studies did not provide adequate data for comparison with other studies
- Studies with unmatched objectives used other OS or LPO markers as a marker of evaluation
- Being literature or systematic reviews
- Duplicated studies (studies involving the same subjects by the same authors)
- Results displayed in histogram representation without sufficient data on MDA levels
- Observational studies involving only pregnant women or children
- The studies analyzed the therapy effect without the control group evaluation
- Studies involving systemic diseases or smoking patients without involving systemically healthy or nonsmoker periodontitis and control group as a separate group of evaluation
- The works have not provided adequate data for comparison with other studies.

### 2.6. Literature Search

First, two authors (K. M. and K. C.) independently searched the electronic database and extracted the relevant studies from all the articles after scanning titles, abstracts, or full texts. Then the identified articles were reviewed using predefined inclusion and exclusion criteria. The articles which did not meet the criteria were excluded from the shortlisted category. In this process, discrepancies were discussed extensively with a third author (C. R.) and resolved. In addition, reference lists from available studies were also screened for other studies that the search strategy had not found.

### 2.7. Evaluation of the Articles

Two observers (S. G. and S. R.) independently evaluated all the studies against the New Castle–Ottawa quality measures for the following criteria: selection of study groups (case definition and representativeness); comparison with the control group (consideration of confounding factors that induce OS such as smoking and systemic diseases); exposure (ethics approval, conflicts of interest statement, interviewer blindness, the similarity between the groups presentation of laboratory determined MDA values and nonresponse rate); other limitations such as imprecision (e.g., inadequate data).

### 2.8. Data Extraction

Two authors (K. M. and C. R.) manually extracted the data from the articles independently. The extracted data from full-text articles were authors, country, year of publication, criteria for periodontitis and control group, sample size, age details of the groups, method of assessment of MDA, MDA values in patients with periodontitis and control group expressed as the mean and SD or median (minimum–maximum) along with specific units and statistical significance. No efforts were made to obtain data that was unavailable from study investigators. The extracted details were reviewed and confirmed by the third author (S. R.) to ensure the accuracy of the collected data, and the discrepancies were discussed to reach a consensus.

### 2.9. Statistical Analyses

Meta-analyses were performed to summarize the differences in LPO biomarker MDA levels between periodontitis patients and healthy controls if three or more studies reported the MDA measurement and expressed by mean ± SD or median (minimum–maximum). Since the selected studies used different assay methods and units for MDA assessment, the standardized mean difference (SMD) and 95% confidence interval (CI) levels were calculated as a summary statistic in meta-analysis to find and analyze the difference in the MDA levels between patients with periodontitis and healthy controls. Regarding the unit of MDA assessment, 1 nmole/ml = 1 *µ*M/l, which is considered a similar unit and considered for meta-analysis. Other than that, no effort was taken for unit or data conversion. The studies were presented with out-of-range values and not presented SD values were not included in the meta-analysis. A random-effects model was used and the included studies were weighed by the generic inverse variance method (Q statistic [17]: *P* < 0.10, *I*^2^ > 50%). The extent of heterogeneity was considered medium to high when the *I*^2^ value was >50%. The statistical analysis was performed by comprehensive meta-analysis software version 3 (Biostat Inc.; Englewood, New Jersey, United States).

## 3. Results

**Figure 1:**
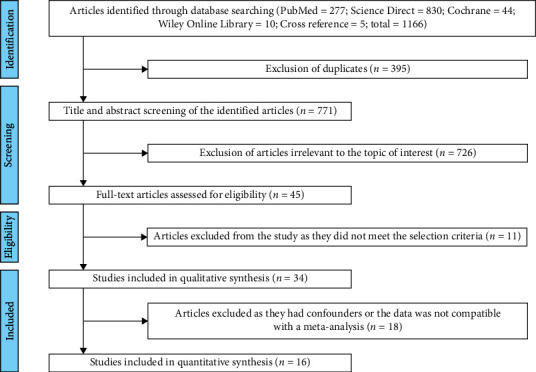
Flowchart for the process of selection of the studies.

The present meta-analysis included all published relevant cross-sectional and case–control studies to provide a comprehensive quantitative synthesis of cumulative evidence. Science Direct search produced 830 articles, PubMed search produced 277 articles, Wiley Online library produced 10 articles, and cross-reference yielded five articles. After the advanced screening of 1,116 articles, 395 were disqualified due to duplicates and 726 were irrelevant to the topic or interest. After the exclusion of these articles, 45 papers had matching objectives to the present SR. The full-text papers were retrieved for the selected articles. Articles with noncoherent data (*n* = 11) were excluded. Only 34 articles were selected after a full-text review according to the exclusion and inclusion criteria for SR. The prospective clinical intervention studies provided the baseline data before therapy were also included in the review. When there were more than two study groups in an individual study, the SR focused only on systemically healthy periodontitis patients and the healthy control group. Studies selected by the investigators for the present SR had an agreement *κ* value of 0.86. Finally, 16 articles had coherent data for meta-analysis. The process of identification and appropriate selection of the studies is described in Figure 1.

In total, 34 articles fulfilled the selection criteria. All included studies had medium or high quality. The measured Cohen's *κ* value of interexaminer reliability was (*κ* = 0.84). The results of the quality scale assessment scale are displayed in Table 1 [18–51].

The studies that obtained scores from 6 to 9 were considered high quality, 3–5 were considered fair quality, and scores from 0 to 2 were considered poor quality. The higher-quality studies were selected for the present SR.

### 3.1. Reasons for Exclusion of Studies after Full-Text Assessment

Five studies with a quality score ≤5 were excluded from the present SR [52–56] during the manuscript revision process by considering the external peer reviewer's suggestions after re-evaluation of the New Castle–Ottawa scale table.

Four studies showed histogram representation for MDA assessment [57–60]. One study evaluated MDA in periodontitis smokers and nonsmokers group without a control group assessment [61]. One study was performed on pregnant women with periodontitis [62].

### 3.2. Data Summary

The summary of all the collected data and assessment methodology in various biological samples from the identified studies [63–75] were displayed in a specified format (Table 2). The measured Cohen's *κ* value of interexaminer reliability was (*κ* = 0.81).

### 3.3. Characteristics of Studies Included in the SR

Most of the studies matched the case and control groups concerning gender and age, mainly with an age range between 25 and 60 years. Nearly 24 studies included individuals with a gingival index (GI) index < 1 as the control group. There were no relevant data about the GI index of the control group in eleven studies. Most of the studies characterized periodontitis by clinical (periodontal pocket depth, clinical attachment level) and radiological assessment (bone loss) factors. Most of the studies utilized the Armitage [76] criteria to diagnose periodontal disease. One study did not mention the criteria used for periodontal disease diagnosis.

Regarding periodontal disease diagnosis, three studies categorized periodontitis into generalized and aggressive periodontitis (AgP) [33, 38, 45]. Two studies categorized periodontitis as early, moderate, and advanced or Stage I and Stage II periodontitis [19, 23]. Three studies included smokers and nonsmokers group patients with periodontitis [22, 24, 27]. One study included the obese and nonobese groups affected with periodontitis [41].

Three studies compared periodontitis and healthy control group with and without systemic diseases such as type II diabetes [34], hyperlipidemia [37], and acute coronary syndrome [39].

Four studies evaluated therapy results with baseline values of the periodontitis and control groups [20, 24, 26, 42].

Two studies assessed TBARs concentration [18, 19], one study assessed LPO concentration [20], and the remaining studies assessed MDA to assess LPO status.

### 3.4. Assessment of MDA in the Included Studies

The detection of thiobarbituric acid reacting substances (TBARS) is a conventional method of MDA assessment, which depends on the reaction with thiobarbituric acid and is detected by spectrophotometric assay [72]. However, this method is not specific to MDA and detects other aldehydes, which also react with thiobarbituric acid and releases a product with similar absorption wavelengths as MDA [80]. TBARS assay still represents a commonly used, cheap, and more accessible method for quantitative measure of LPO. Some advanced, more reliable, and specific methods for measuring MDA are liquid chromatography and mass spectroscopy [21].

The present SR systematically summarized the results of 34 independent studies involving 1,342 patients with periodontitis and 967 healthy controls from different countries. Out of 34 studies of MDA assessment in various samples, nearly 17 studies confirmed significantly (*P* < 0.001) higher MDA levels in patients with periodontitis compared with the clinically healthy control group, which indicated an elevation in OS status in periodontitis patients (Table 2).

### 3.5. Meta-Analysis

**Figure 2:**
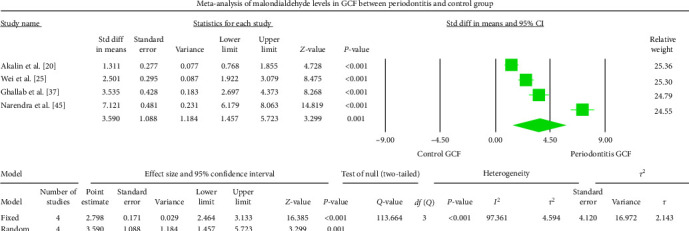
The Forest plot displays SMD values with a confidence interval of 95%, representing the differences in GCF MDA levels between the patients with periodontitis and the healthy group.

**Figure 3:**
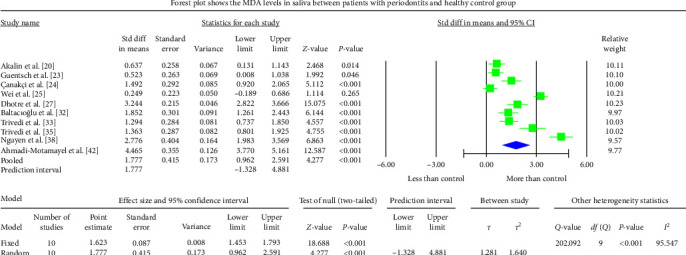
The Forest plot displays SMD values with a confidence interval of 95%, representing the differences in salivary MDA levels between patients with periodontitis and the healthy group.

**Figure 4:**
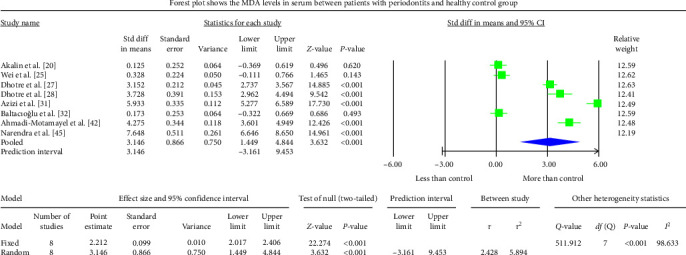
The Forest plot displays SMD values with a confidence interval of 95%, representing the differences in serum MDA levels between patients with periodontitis and the healthy group.

The periodontitis group displayed significantly higher MDA levels (*P* < 0.001) in GCF, saliva, and serum samples of the studies analyzed. The GCF samples depicted an overall standardized mean difference MDA value of 3.590 nmol/l (95% CI: 1.457–5.723) (Figure 2). The salivary samples showed an overall standardized mean difference MDA value of 1.777 nmol/l (95% CI: 0.962–2.591) (Figure 3). The serum samples displayed an overall standardized mean difference MDA value of 3.146 nmol/l (95% CI: 1.449–4.844) (Figure 4). The analysis of MDA values after therapeutic intervention could not be achieved due to the scarcity of published reports.

The meta-analysis of the MDA assessment between periodontitis patients and the healthy control group displayed high heterogeneity, which was reflected by the greater *I*^2^ values of 97.361, 95.547, and 98.633 in Figures 2–4, respectively. The reasons for such variability are both technical and biological. The different methodologies and protocols utilized to measure MDA values could have caused higher heterogeneity. The heterogeneity may also be induced by a different population of periodontitis (sex and age) and different biological specimens (GCF, saliva, serum, plasma, or whole blood) among different studies and different study designs (cross-sectional, case–control, or interventional). The SMD effect scale was used to reduce the discrepancy and the random-effects model was applied in the meta-analysis.

### 3.6. Publication Bias

Studies included in the meta-analysis of MDA assessment between patients with periodontitis and the healthy group showed Egger's regression intercept values of 21.750 and 8.177 with two-tailed *P*-values 0.09 and 0.37 in GCF and saliva samples, respectively, indicating a lower risk of publication bias of selected studies in the present meta-analysis. Begg and Mazumdar's test for rank correlation denoted a *P*-value of 0.137, indicating no risk of publication bias for included studies of meta-analysis in salivary samples.

## 4. Discussion

The imbalance of ROS and antioxidant systems leads to OS, which contributes to functional and structural remodeling that favors the occurrence of periodontitis. Some studies pointed out ROS production by inflammatory neutrophils [81] and others described that ROS actively released by micro-organisms might contribute to OS in periodontitis [5]. ROS causes tissue damage via multiple mechanisms, including DNA damage, LPO damage, and enzyme oxidation [7, 21]. The end products of these OS-mediated reactions of cellular biomolecules can be used as biomarkers of OS-associated periodontitis. LPO destroys cellular membrane lipids and initiates a pathway of the oxidation of PUFA, ultimately synthesizing MDA by maintaining through chain reactions. MDA, which can indicate the status of OS, is the primary and most stable product of PUFA peroxidation. MDA is a commonly measured LPO product to indicate OS in various diseases, including periodontitis [43].

Remarkably, many observational studies analyzing OS had relatively consistently elevated LPO end product MDA in patients with periodontitis compared with controls.

All the reported studies in the present SR that evaluated GCF MDA levels depicted significantly higher levels in periodontitis patients than in healthy controls. The meta-analysis of GCF samples depicted an overall standardized mean difference MDA value of 3.590 nmol/l (95% CI: 1.457–5.723) when the periodontitis group compared with the healthy control group.

Salivary MDA values in periodontitis were extensively investigated. Most studies showed higher salivary MDA levels in periodontitis patients than in healthy controls, except for one report [53]. Shankarram et al. [55] reported insignificant differences in salivary MDA levels between patients with periodontitis and healthy controls. The salivary samples showed an overall standardized mean difference MDA value of 1.777 nmol/l (95% CI: 0.962–2.591) when the periodontitis group was compared with the healthy control group. The increased GCF or salivary MDA level could have resulted from superoxide anion production during the interaction with periodontal pathogens or the by-product with neutrophils within periodontal tissues or pockets. These results suggest that salivary or GCF MDA levels could also be used to indicate periodontal damage by ROS.

There are also few studies investigating the level of MDA in the serum and saliva of periodontitis patients; their results were controversial. Though significantly higher MDA levels were observed in salivary samples, the differences were insignificant in serum samples [21, 33, 42, 45]. Their finding suggests that the effect of periodontitis on systemic OS might be limited. However, Wei et al. [26] reported insignificant MDA level differences between periodontitis and healthy controls in salivary and serum samples. The remaining studies of the present SR found significantly higher serum or plasma MDA in periodontitis than in healthy controls [27–30, 32, 34, 40, 43, 46, 49, 51, 54, 56]. The serum samples displayed an overall standardized mean difference MDA value of 3.146 nmol/l (95% CI: 1.449–4.844) when the periodontitis group was compared with the healthy control group.

Few studies confirmed the positive correlation between these markers with periodontal status scores [7, 82]. Baltacıoğlu et al. [33] compared salivary MDA and healthy controls and found that periodontitis and AgP groups have significantly higher MDA levels than the control group. However, no differences between AgP and periodontitis groups were observed. Another study by Ghallab et al. [38] demonstrated that levels of MDA in GCF could differentiate between general periodontitis, AgP, and periodontally healthy controls. Other studies found higher LPO in patients with severe but not moderate periodontitis [19, 23]. It has also been displayed that the higher levels of MDA in patients with periodontitis can be diminished after periodontal therapy [20, 24, 26].

An important secondary finding was a positive correlation between LPO in saliva and GCF [20]. In contrast, Celec et al. [83] reported no specific association observed between plasma and salivary TBARS values in periodontitis. Baňasová et al. [59] study reported significantly higher salivary TBARS in male patients with periodontitis than the healthy controls, but this is not the case in female patients. That could be due to changes in salivary cytokines during the menstrual cycle [84].

Meanwhile, studies including diabetes mellitus, acute coronary syndrome, and hyperlipidemia pointed out that periodontitis could contribute to the higher systemic level of MDA among patients with systemic pathologies [34, 37, 39].

Conclusively LPO biomarker MDA mean values of various samples significantly differed between periodontitis patients and healthy subjects. It has been confirmed that elevated ROS production by the inflammatory cells in periodontitis is associated with increased local and systemic OS, which promotes tissue destruction in periodontal disease. More importantly, the status of OS parameters in biological samples can reflect their association with periodontal disease.

## 5. Conclusion

Our meta-analysis results suggested that LPO biomarker MDA levels from various biological samples were significantly different between patients with periodontitis and healthy subjects. Despite the limitations of the present meta-analysis, the results supported the fact that there was a direct association between periodontitis and LPO-related biomarkers levels, indicating the critical role of OS in periodontal disease.

## Figures and Tables

**Table 1:** New Castle Ottawa scale for the articles included in the systematic review assessment.

Study (reference)	Selection				Comparability		Exposure				Total score
	Case definition	Case representativeness	Control selection	Control definition	Matching known confounding factor	Matching potential confounding factor	Secure patient records	Interviewer blinded to cases and control	Similarity in the case and control ascertainment	Non-response rate	
Panjamurthy et al. [18]	+	+	+	+	+	+	+	−	−	−	7
Mashayekhi et al. [19]	+	+	+	+	+	+	+	−	+	−	8
Tsai et al. [20]	+	+	+	+	+	+	+	−	+	−	8
Akalin et al. [21]	+	+	+	+	+	+	+	−	+	−	8
Borges et al. [22]	+	+	+	+	+	+	+	−	+	−	8
Khalili and Biloklytska [23]	+	+	+	+	+	+	+	−	+	−	8
Guentsch et al. [24]	+	+	+	+	+	+	+	−	+	+	9
Çanakçi et al. [25]	+	+	+	+	+	+	+	−	+	−	8
Wei et al. [26]	+	+	+	+	+	+	+	−	+	−	8
Tonguç et al. [27]	+	+	+	+	+	+	+	−	+	−	8
Dhotre et al. [28]	+	+	+	+	+	+	+	−	+	−	8
Dhotre et al. [29]	+	+	+	+	+	+	+	−	+	−	8
Gupta et al. [30]	+	+	+	+	+	+	+	−	+	−	8
Miricescu et al. [31]	+	+	+	+	+	+	+	−	+	−	8
Azizi et al. [32]	+	+	+	+	+	+	+	−	+	−	8
Baltacıoğlu et al. [33]	+	+	+	+	+	+	+	−	+	−	8
Trivedi et al. [34]	+	+	+	+	+	+	+	−	+	−	8
Almerich-Silla et al. [35]	+	+	+	+	+	−	−	−	+	−	6
Trivedi et al. [36]	+	+	+	+	+	+	+	−	+	−	8
Fentoğlu et al. [37]	+	+	+	+	+	+	+	−	+	−	8
Ghallab et al. [38]	+	+	+	+	+	+	+	−	+	−	8
Nguyen et al. [39]	+	+	+	+	+	+	+	−	+	−	8
Dahiya et al. [40]	+	+	+	+	+	+	−	−	+	−	7
Atabay et al. [41]	+	+	+	+	+	+	+	+	+	−	9
Önder et al. [42]	+	+	+	+	+	+	+	−	+	−	8
Ahmadi-Motamayel et al. [43]	+	+	+	+	+	+	+	−	+	−	8
Lutfioğlu et al. [44]	+	+	+	+	+	+	+	+	+	−	9
Tripathi et al. [45]	+	+	+	+	+	+	+	−	+	−	8
Narendra et al. [46]	+	+	+	+	+	+	+	−	+	−	8
Cherian et al. [47]	+	+	+	+	+	+	+	−	+	−	8
Sánchez-Villamil et al. [48]	+	+	+	+	+	+	+	−	+	−	8
Inasu et al. [49]	+	+	+	+	+	+	+	−	+	−	8
Warad et al. [50]	+	+	+	+	+	+	+	−	+	−	8
Veljovic et al. [51]	+	+	+	+	+	+	+	−	+	+	9

**Table 2:** The mean and SD values of MDA in various biological fluid samples between healthy groups and patients with periodontitis in the selected studies of qualitative synthesis.

Study name	Country	Type	Case criteria (periodontitis)	Age (years) group PD/control (mean ± SD or median, minimum–maximum)	Sample type	Unit of measurement	Case mean ± SD/median (minimum–maximum)	Case sample size (M/F)	Control mean ± SD	Control sample size (M/F)	*P*-value	Method
Panjamurthy et al. [18]	India	CC	PPD (>3.5 mm), gingival recession (Grade III), furcation involvement and degree of tooth mobility.	25–35 (both groups)	Plasma TBARs	nmol/ml	10.34 ± 1.9	25 M	4.79 ± 0.83	25 M	<0.001	Yagi [63]
					RBC	pmol/mg Hb	8.79 ± 2.1	25 M	3.65 ± 0.52	25 M	<0.001	Donnan [64]
					RBC memb	nmol/mg protein	1.06 ± 0.13	25 M	0.39 ± 0.06	25 M	<0.001	
					Gingival tissue	nmol/100 mg protein	170.6 ± 24.8	25 M	130.8 ± 16.9	25 M	<0.001	Ohkawa et al. [65]

Mashayekhi et al. [19]	Iran	CC	American Dental Association	25–55 (both groups)	Saliva TBARs	*μ*mol/ml	Early (1.22); moderate (1.22); advanced (1.35)	24 (12/12) early (8); moderate (8); advanced (8)	1.2	8	<0.01-Advanced	Satoh [66]

Tsai et al. [20]	Taiwan	CC	PPD ≥ 3 mm, PAL ≥ 2 mm, and GI ≥ 1		Saliva LPO	*µ*M	0.66 ± 0.36	13	0.13 ± 0.08	9	<0.0005	Bioxy tech assay
					GCF LPO	*µ*M	151.9 ± 77.7	13	50.66 ± 37.22	9	<0.005	

Akalin et al. [21]	Turkey	CC	AAP [76]: 30% periodontal bone loss with PAL ≥ 2 mm and GI ≥ 5 mm deep pockets with bleeding on probing	40.66 ± 5.31/38.5 ± 6.10	Saliva	*µ*M	0.127 ± 0.08	36 (19/17)	0.08 ± 0.059	28 (13/15)	<0.05	Young and Trimble [67]
				40.66 ± 5.31/38.5 ± 6.10	Serum	*µ*M	0.6 ± 0.16	36 (19/17)	0.58 ± 0.16	28 (13/15)	>0.05	
				40.66 ± 5.31/38.5 ± 6.10	GCF	*µ*M	0.88 ± 0.18	36 (19/17)	0.67 ± 0.13	28 (13/15)	<0.05	

Borges et al. [22]	Brasil	CC	BOP: at least five or six sites with PPD ≥ 5 mm, attachment loss ≥3 mm, and extensive radiographic bone loss	52.9 ± 5.0/51.1 ± 9.6	Gingival tissue TBARS	nmol/g protein	188.8 ± 20.7	9 (4/5)	113.1 ± 16.59	9 (4/5)	0.015	Ohkawa et al. [65]

Khalili and Biloklytska [23]	Ukraine	CC	AAP [78]: CAL, early (1–2 mm), moderate (3–4 mm), and severe (≥5 mm) with ≥30% of the sites	18–65/22–29 years	Saliva	*μ*mol/l	Early (28.08 ± 1.56); moderate (39.01 ± 1.59); severe (65.20 ± 2.00)	74 (22/52) early (30); moderate(30); severe (14)	5.16 ± 0.03	30 (10/20)	<0.05	Stalnaya and Garishvili [68]

Guentsch et al. [24]	Germany	PS	At least 30% of teeth with pockets >5 mm	46.3 ± 13.1/34.1 ± 11.8	Saliva	*μ*mol/l	0.109 ± 0.07	30 (14/16)	0.075 ± 0.065	30 (14/16)	<0.05	Yagi [69]

Çanakçi et al. [25]	Turkey	CS	AAP [76]: two sites with PPD of ≥4 mm, gingivitis and 30% bone loss	45.3 ± 0.97/42.7 ± 12.4	Saliva	nmol/ml	7.35 ± 1.45	30 (15/15)	5.41 ± 1.13	30 (15/15)	<0.001	Jain et al. [70]

Wei et al. [26]	China	PS	AAP [76]: teeth with 30% periodontal bone loss and with ≥5 mm deep pockets with gingivitis and bleeding on probing	40.1 ± 7.3/42.1 ± 7.7	Saliva	mmol	0.11 ± 0.05	48 (27/21)	0.1 ± 0.02	35 (19/16)	>0.05	Young and Trimble [67]
				40.1 ± 7.3/42.1 ± 7.7	Serum	mmol	0.72 ± 0.13	48 (27/21)	0.68 ± 0.11	35 (19/16)	>0.05	
				40.1 ± 7.3/42.1 ± 7.7	GCF	mmol	1.03 ± 0.22	48 (27/21)	0.51 ± 0.19	35 (19/16)	<0.05	

Tonguç et al. [27]	Turkey	CS	Armitage [76]: moderate generalized periodontitis (≥3 mm AL, <5 mm throughout ≥30% of the mouth)	20–50/25–49	Gingival tissue	nmol/mg protein	1.257 ± 0.49	65 (32/33)	0.863 ± 0.759	20 (11/9)	<0.01	Draper and Hadley [71]
				20–50/25–49	Blood	nmol/mg hemoglobin	77.2 ± 81.8	65 (32/33)	40.2 ± 14.27	20 (11/9)	<0.01	

Dhotre et al. [28]	India	CC	AAP [76]: teeth with 30% periodontal bone loss and with ≥5 mm deep pockets	52.7 ± 9.27/50.3 ± 9.39	Saliva	nmol/ml	7.36 ± 0.72	100 (60/40)	5.21 ± 0.6	100 (60/40)	<0.001	Satoh [66]
				52.7 ± 9.27/50.3 ± 9.39	Serum	nmol/ml	6.55 ± 1.21	100 (60/40)	3.71 ± 0.4	100 (60/40)	<0.001	

Dhotre et al. [29]	India	CC	CAL ≥ 4 mm, PPD ≥ 4 mm, with BOP		Serum	nmol/ml	6.97 ± 1.03	50	3.71 ± 0.4	25	<0.001	Satoh [66]

Gupta et al. [30]	India	CC	Existence of calculus and plaques with attachment loss of ≥2 mm in at least three different sites	30–65 (case)	Saliva	nmol/l	3.39 ± 0.54	30	1.17 ± 0.27	30	<0.001	Randox laboratory
				30–65 (case)	Serum	nmol/l	1.75 ± 0.35	30	1.18 ± 0.28	30	<0.001	

Miricescu et al. [31]	Romania	CC	At least six sites with PPD ≥ 4 mm, LB > 30% with gingivitis	51.26 ± 7.4/18.66 ± 2	Saliva	nmol/mg albumin	0.296 ± 0.1	25 (14/11)	0.25 ± 0.4	25 (20/5)	<0.05	TBARS assay

Azizi et al. [32]	India	CC	AAP [76]: at least 30% sites with PPD 5 mm and CAL ≥ 3 mm with gingivitis and BOP	37–50/39.64 ± 5.04	Serum	nmol/ml	4.4 ± 0.45	134 (M)	2.1 ± 0.2	64 (M)	<0.001	Satoh [66]

Baltacıoğlu et al. [33]	Turkey	CS & CC	AAP [76]: multiple sites with PPD ≥ 5 mm, LB ≥ 30%, and LA ≥ 5 mm	32.55 ± 5.32/30.10 ± 4.06	Saliva	*µ*mol	Periodontitis: 0.15 (0.1–0.18); GAP: 0.15 (0.14–0.18)	33 (16/17) periodontitis; 35 GAP	0.087 ± 0.035	30 (16/14)	<0.05	Young and Trimble [67]
				32.55 ± 5.32/30.10 ± 4.06	Serum	*µ*mol	Periodontitis: 0.62 ± 0.14; GAP: 0.63 ± 0.09	33 (16/17) periodontitis; 35 GAP	0.6 ± 0.08	30 (16/14)	>0.05	

Trivedi et al. [34]	India	CS & CC	Armitage [76]: two or more tooth sites with PP ≥ 4 mm or CAL ≥ 4 mm that bled on probing	20–65	Saliva	nmol/ml	9.09 ± 8.16	30 (11/19)	1.53 ± 1.3	30 (6/24)	<0.05	Ohkawa et al. [65]
				20–65	Plasma	nmol/ml	17.56 ± 6.07	30 (11/19)	8.62 ± 8.46	30 (6/24)	<0.05	

Almerich-Silla et al. [35]	Spain	CC	At least four zones with PP ≥ 5 mm and LA ≥ 2 mm	41–45/38–43	Saliva	nmol	5.94 ± 1.02	33 (19/14)	4.427 ± 0.922	37 (15/22)	<0.05	NWLSS; NWK-MDA01

Trivedi et al. [36]	India	CS &CC	Armitage [76]: at least 30% sites with pockets >5 mm	30.7 ± 5.3/35.7 ± 5.9	Saliva	nmol/ml	9.34 ± 8.15	30 (14/16)	1.39 ± 1.28	30 (15/15)	<0.001	Ohkawa et al. [65]

Fentoğlu et al. [37]	Turkey	CC	Armitage [76]: ≥4 teeth with PD ≥ 5 mm and CAL ≥ 2 mm at the same time	43.47 ± 9.53/43.41 ± 9.82	Serum	nmol/g	31.64 ± 11.51	19 (9/10)	39.11 ± 10.01	19 (9/10)	<0.04	Draper and Hadley [71]

Ghallab et al. [38]	Egypt	CS	AAP [77]: minimum of six teeth with at least one site each with PPD and CAL > 5 mm	42.2 ± 2.55/26 ± 2.67	GCF	*µ*mol	Periodontitis: 1.1 ± 0.2; GAP: 1.8 ± 0.4	25 (14/11)	0.5 ± 0.1	15 (6/7)	<0.001	Young and Trimble [67]

Nguyen et al. [39]	Vietnam	CS	Armitage [76]: at least 10 sites with a PPD of ≥5 mm and >30% of the sites with an AL of ≥2 mm	51.04 ± 12.25/51.17 ± 11.88	Saliva	*µ*mol	1.55 ± 0.52	24 (12/12)	0.36 ± 0.44	24 (14/10)	<0.05	Ohkawa et al. [65]

Dahiya et al. [40]	India	CC	AAP [76]: at least with 30% periodontal bone loss and with ≥5 mm deep pockets	20–50 (both groups)	Serum	nmol/dl	308 ± 58.5	20 (10/10)	194.4 ± 22	20 (10/10)	<0.01	Yagi [72]

Atabay et al. [41]	Turkey	CC	Armitage [76]: ≥30% of sites with PPD ≥ 5 mm and with CAL ≥ 5 mm and ≥30% alveolar bone loss	42.47 ± 2.99/39.60 ± 5.84	GCF	*µ*mol/l	826.4 ± 79	15 (9/6)	566.3 ± 14.1	15 (6/9)	<0.001	Bioxytech, MDA-586

Önder et al. [42]	Turkey	PS	Armitage [76]; minimum of 16 teeth and eight sites with PPD ≥ 6 mm, and at least four sites with AL ≥ 5 mm in two quadrants	45.8 ± 5.2/45.1 ± 6.6	Serum	pmol/l	0.165 ± 0.01	25 (14/11)	0.16 ± 0.016	26 (11/15)	<0.612	Canakci et al. [73]
				45.8 ± 5.2/45.1 ± 6.6	Saliva	pmol/ml	0.602 ± 0.18	25 (14/11)	0.355 ± 0.1	26 (11/15)	<0.001	

Ahmadi-Motamayel et al. [43]	Iran	CC	CAL of ≥4 mm in two or more teeth [79]	30–50 (both groups)	Serum TBARs	nmol/ml	1.76 ± 0.09	55 (28/27)	1.15 ± 0.18	56 (28/28)	0.0001	Rai et al. [52]
				30–50 (both groups)	Saliva	nmol/ml	0.8 ± 0.09	55 (28/27)	0.42 ± 0.08	56 (28/28)	0.0001	

Lutfioğlu et al. [44]	Turkey	CC	PI: 2.10 ± 0.18, GI: 2.31 ± 0.40, BOP: 92.72 ± 10.2%, PPD: 5.30 ± 0.55 mm, CAL: 7.41 ± 0.88 mm	42.17 ± 3.1/38.30 ± 4.84	GCF	*μ*mol	802.1 ± 88.2	15 (9/6)	144.1 ± 4.15	15 (7/8)	<0.01	Bioxy tech, MDA-586

Tripathi et al. [45]	India	CC	≥30% of periodontal bone loss, with CL of ≥5 mm, along with PPD of ≥5 mm at more than one site of all tooth quadrants		Saliva	*µ*mol	Periodontitis: 0.16; GAP: 0.16	40	0.07	40	0.04	Young and Trimble [67]
					Serum	*µ*mol	Periodontitis: 0.68; GAP: 0.65	40	0.61	40	0.336	

Narendra et al. [46]	India	CC	AAP [76]: PPD ≥ 4 mm in more than 1/3rd of the total teeth, with BOP	47.13 ± 7.00/36.56 ± 6.26	Serum	nmol/ml	2.02 ± 0.32	46 (29/17)	0.59 ± 0.14	50 (33/17)	<0.001	Satoh [66]
				47.13 ± 7.00/36.56 ± 6.26	GCF	nmol/ml	1.98 ± 0.32	46 (29/17)	0.63 ± 0.12	50 (33/17)	<0.001	

Cherian et al. [47]	India	CC	At least four teeth with one or more sites exhibiting CAL ≥ 4 mm, PPD ≥ 4 mm, and BOP	18–45	Saliva	*µ*mol/100 ml	281.6 ± 83.5	30	89.45 ± 46.47	30	<0.001	Esa [74]

Sánchez-Villamil et al. [48]	Colombia	CS & CC	Page and Eke [79]: ≥2 interproximal sites with CAL ≥ 3 mm, PPD ≥ 4 mm (not in the same tooth)	45 ± 12/31 ± 10	Saliva	*µ*mol/g protein	2.1 ± 1.54	87 (45/42)	0.46 ± 0.3	14 (6/8)	<0.0001	Sigma–Aldrich assay

Inasu et al. [49]	India	CC	1999 International Workshop: more than 30% of sites with CAL > 3 mm and PPD > 4 mm		Serum	*µ*mol/l	1.791 ± 0.89	30	0.847 ± 0.461	30	<0.001	Buege and Aust [75]
					Saliva	*µ*mol/l	1.783 ± 0.45	30	0.485 ± 0.292	30	<0.001	

Warad et al. [50]	India	CC	1999 International Workshop: at least 30% teeth with PPD > 5 mm and CAL > 4 mm		Saliva	*µ*mol/ml	8.96 ± 2.59	30	5.42 ± 1.55	30	<0.0001	Stalnaya and Garishvili [68]

Veljovic et al. [51]	Serbia	CC	≥2 sites per quadrant with PD ≥ 4 mm, ≥30% bone loss and gingivitis	48.70 ± 9.68/46.25 ± 9.25	Saliva	pmol/*µ*l	2.99 ± 1.21	30 (10/20)	1.33 ± 0.92	20 (9/11)	<0.05	ELISA kit, Cell Biolabs
				48.70 ± 9.68/46.25 ± 9.25	Plasma	pmol/*µ*l	0.5 ± 0.13	30 (10/20)	0.4 ± 0.13	20 (9/11)	<0.05	

Case, periodontitis; GAP, generealized aggressive periodontitis; SD, standard deviation; CC, case–control; CS, cross-sectional; PS, prospective; TBARs, thiobarbituric acid reacting substances; MDA, malondialdehyde; LPO, lipid peroxidantion products; RBC, red blood cells; RBC memb, RBC membrane; GCF, gingival crevicular fluid; HPLC, high-performance liquid chromatography; MDA, malondialdehyde; LPO, lipid peroxidation; GI, gingival index; CAL/CL, clinical attachment level; PD/PPD, pocket depth or probing depth; PP, periodontal pocket; LA, loss of attachment; PAL, periodontal attachment level; LB, level of bone loss; BOP, bleeding on probing; NWLSS, Northwest Life Science Specialities; AAP, American Academy of Periodontology.

## Data Availability

Data analyzed in this study were a reanalysis of existing data, which are openly available at locations cited in the reference section.

## Abbreviations

MDA: Malondialdehyde
MeSH: Medical subject headings
GCF: Gingival crevicular fluid
ROS/RNS: Reactive oxygen species/reactive nitrogen species
DNA: Deoxyribonucleic acid
OS: Oxidative stress
LPO: Lipid peroxidation
SR: Systematic review
SD: Standard deviation
SMD: Standardized mean difference
CI: Confidence interval
TBARs: Thiobarbituric acid reacting substances
AgP: Aggressive periodontitis
PPD: Periodontal pocket depth
CAL: Clinical attachment level
GI: Gingival index.

## Additional Points

*Limitations*. Different measurement methods were utilized in different studies to detect LPO markers, so the MDA levels were in different orders of magnitude in reported studies. The heterogeneity persisted even though the pooled studies displayed measurements with reasonably comparable methods. The above discrepancies might also be due to the differences in patient populations, sample size, laboratory detection methods, and biological specimens used for OS biomarker detection.
